# Runx1-Stat3 signaling regulates the epithelial stem cells in continuously growing incisors

**DOI:** 10.1038/s41598-018-29317-6

**Published:** 2018-07-19

**Authors:** Safiye E. Sarper, Toshihiro Inubushi, Hiroshi Kurosaka, Hitomi Ono Minagi, Koh-ichi Kuremoto, Takayoshi Sakai, Ichiro Taniuchi, Takashi Yamashiro

**Affiliations:** 1 Department of Orthodontics and Dentofacial Orthopedics, Osaka University Graduate School of Dentistry, Osaka, Japan; 2 Department of Oral-facial Disorders, Osaka University Graduate School of Dentistry, Osaka, Japan; 3 Department of Advanced Prosthodontics, Graduate School of Biomedical & Health Sciences, Hiroshima University, Hiroshima, Japan; 4 Laboratory for Transcriptional Regulation, RIKEN Research Center for Allergy and Immunology, Yokohama, Japan

## Abstract

Rodent incisors grow permanently and the homeostasis of enamel production is maintained by a continuous supply of epithelial progenitors from putative stem cells in the cervical loop. We herein report that Runx1 regulates the *Lgr5*-expressing epithelial stem cells and their subsequent continuous differentiation into ameloblasts. Mice deficient in epithelial Runx1 demonstrate remarkable shortening of the incisors with underdevelopment of the cervical loop and enamel defects. In this mutant cervical loop, the proliferation of the dental epithelium was significantly disturbed and the expression of *Lgr5* and enamel matrix proteins was remarkably downregulated. Interestingly, the expression of *Socs3*, an inhibitor of Stat3 signaling, was upregulated and Stat3 phosphorylation was suppressed specifically in the mutant cervical loop. The expression of *Lgr5* and the enamel matrix protein in the wild-type incisor germs is disturbed by pharmaceutical Stat3 inhibition *in vitro*., of. Conversely, pharmaceutical activation of Stat3 rescues the defective phenotypes of the *Runx1* mutant with upregulated *Lgr5* and enamel matrix protein genes. The present results provide the first evidence of the role of Runx1 regulates the *Lgr5*-expressing epithelial stem cells and differentiation of ameloblast progenitors in the developing incisors. Our study also demonstrates that Stat3 modulates the Runx1-Lgr5 axis in the cervical loop.

## Introduction

The rodent incisor represents a special type of tooth, as it grows continuously throughout the lifetime of the animal. Its regenerative capacity implies the presence of a stem cell pool^1^. The cervical loop, which is located at the proximal end of the incisor, has been shown to contain the epithelial stem cell niche that houses the progenitors of ameloblasts, which secrete enamel matrix at the labial surface of the incisors^2,3^. Many signaling molecules and growth factors have been implicated in the mediation of these interactions^4^, and several molecules are specifically involved in the maintenance of the continuous regeneration of the incisors^5^.

The Runx family genes are important transcriptional regulators in the embryonic development of various tissues and the pathogenesis of various diseases. Among the Runx family genes, Runx1 is recognized as a human hematopoietic stem cell factor^6^. In addition, Runx1 is also important for skin stem cells as well as skin cancer development. Furthermore, the epithelial-targeted deletion of *Runx1* affects the hair shaft structure and the homeostasis of hair follicle stem cells^7,8^. On the other hand, our previous study demonstrated that the cervical loop did not fully develop in the incisors of mice deficient in epithelial *Cbfb*, which resulted in short incisors^9^. Cbfb is a non-DNA binding regulatory subunit and forms a heterodimer with the Runx proteins, as the complex works as a transcription factor^10^, However, the specific alpha unit of the RUNX protein that is required in the development of the incisors has remained unclear. In addition, the molecular mechanism underlying the regulation of stem cells in the incisors by Runx signaling is still largely unknown.

In mammals, JAK/STAT signaling is essential for a wide array of cytokines and growth factors^11,12^. Upon activation, phosphorylated Stats dimerize and translocate to the nucleus where they modulate the expression of target genes. Among the various functions of the Stat family, Stat3 is involved in the regulation of the self-renewal of embryonic stem cells, cancer stem cells, hematopoietic stem cells and the stem cells in hair follicles. Indeed, Lif activates Stat3 activation in ES cells^13^. In hair growth, the depletion of stat3 results in decreases in the stem cell colony and prolongs the hair regeneration cycle^14^. In hair development, Stat3 activity is regulated by Runx1 and the depletion of *Runx1* leads to the inhibition of Stat3 phosphorylation in the bulge of the hair follicle and results in hair cycle dysregulation^7,15^. In addition, the overexpression of *Runx1* leads to Stat3 activation and is necessary for the regulation of cellular proliferation and tumorigenesis in the skin^16^. STAT3 is involved in human breast cancer, in which high STAT3 levels are correlated with a poorer survival^17^. However, the possible involvement of Stat3 in the stem cell homeostasis in the growing incisors has not been investigated.

Lgr5 is a general stem cell marker and has been identified as a Wnt target genes in the intestinal stem cell niche^18,19^. In the growing incisors, the expression of *Lgr5* is localized in the stem cell compartment in the cervical loop epithelium^20,21^. However, canonical Wnt signaling is exclusively evident in the mesenchymal tissue of the reporter mice, but not in the cervical loop epithelium^22^. On the other hand, Fgf10/Fgfr2b signaling plays essential roles in the regulation of the cervical loop stem cells^1,20,21,23^. In this signaling axis, the inhibition of FGFR or its downstream signal transduction pathways was shown to diminish the *Lgr5*-expressing cells in the cervical loop^24^. However, the molecular mechanism through which the *Lgr5* expression is regulated in the cervical loop remains to be elucidated.

We herein report that Runx1 is involved in the regulation of epithelial stem cells and their subsequent differentiation into ameloblasts in the rodent incisors, using mice with epithelial-specific deletion of *Runx1*. We show that *Runx1* deficiency results in the marked shortening of the incisors with the underdevelopment of the cervical loop. Indeed, the expression levels of *Lgr5* and *Sox2* (general stem cell markers) are significantly downregulated due to *Runx1* deficiency. *Runx1* mutant mice also show enamel defects with the significant downregulation of *amelogenin* and *ameloblastin* (ameloblast differentiation markers). We show that Stat3 phosphorylation of the mutant incisors is disturbed by *Runx1* deficiency, specifically in the cervical loop epithelium. Indeed, the application of Stat3 inhibitors remarkably suppressed the expression of *Lgr5* and enamel matrix protein of explanted incisors in *in vitro* culture. Conversely, treatment with Stat3 activator partially rescued the suppressed *Lgr5* and *Amelogenin* expression in *Runx1* mutant incisors in culture. Thus, we demonstrate that Runx1 is involved in the regulation of *Lgr5*-positive epithelial stem cells and ameloblast progenitors, at least in part, through the regulation of Stat3 phosphorylation in the cervical loop of the growing incisors.

## Results

### The expression of Runxs mRNA in the growing incisors

**Figure 1:**
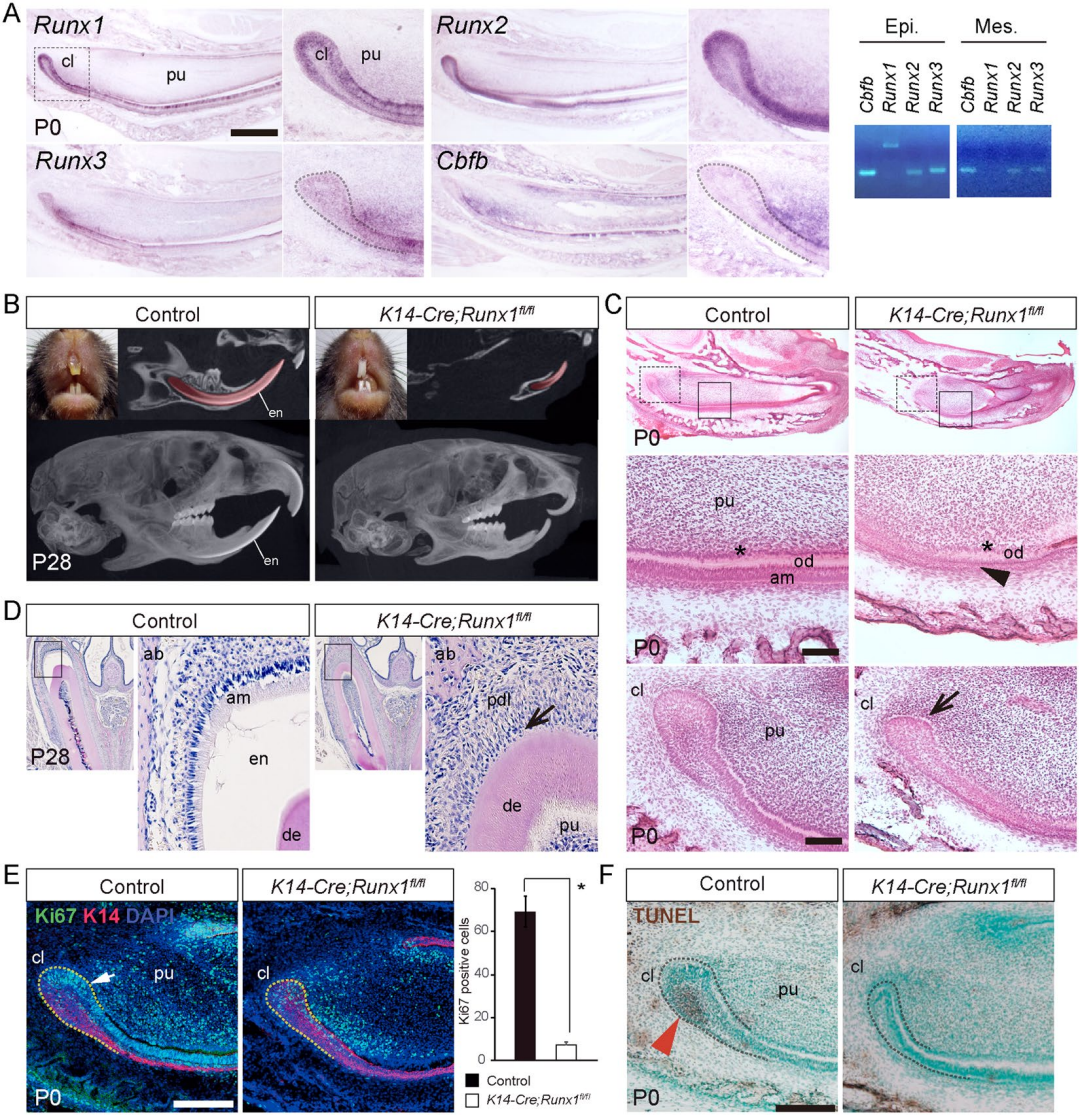
The incisor phenotype of *K14-Cre/Runx1*^*fl/fl*^ mice. (**A**) Expression of *Runx1, Runx2, Runx3* and *Cbfb* in sagittal sections of P0 incisors and gene expression was confirmed by RT-PCR from dissected epithelium and mesenchyme. *Runx1* transcripts were detected in the dental epithelium. In the cervical loop, *Runx1* was expressed in the basal epithelial cells (inner enamel epithelium) and the outer enamel epithelium in the cervical loop. Scale bar: 500 μm. (**B**) The *Runx1* mutant incisors demonstrated a column shape and lost their transparency, although the control incisors developed fine edged shape. In the micro-CT analysis, the incisors of the *Runx1* mutant mice had an abnormal shape, extreme shortening and enamel defects. On the other hand, significant differences were not detected in the molar root length or morphology. (**C**) HE staining of incisor sagittal sections from P0 *Runx1* mutant mice confirmed the shortening of the *Runx1* mutant incisors. At higher magnification, hypoplasia of the cervical loop (arrow) was evident and ameloblast precursors lost their polarity and had a flattened shape in *Runx1* mutants (arrowhead). Scale bar: 100 μm. (**D**) HE staining of incisor frontal sections from P28 *Runx1* mutant mice confirmed the lack of enamel matrix formation. At higher magnification, enamel matrix was not evident between the dentin and the periodontal ligament (arrow) in the upper incisors of *Runx1* mutants. (**E**) Double immunostaining for Ki67 (green) and K14 (red) revealed that the epithelium in the cervical loop of the *Runx1* mutant was significantly less proliferative (arrow). Nuclei were counterstained with DAPI (blue). Scale bar: 200 μm. (**F**) TUNEL staining revealed that the number of TUNEL-positive cells was significantly reduced at the cervical loop of the Runx1 mutant incisors (arrowhead). cl, cervical loop; pu, dental pulp; en, enamel; am, ameloblasts; od, odontoblasts. Scale bar: 200 μm.

The previous study reported that *Runx1* mRNA is expressed in the dental epithelium^25^. The present *in situ* hybridization analysis revealed that *Runx1* was present in the basal epithelial cells (inner enamel epithelium) and the outer enamel epithelium in the cervical loop at P0 (Fig. 1A). RT-PCR analysis demonstrated that *Runx1* expression was not detected in the dental mesenchyme. *Runx2* mRNA demonstrated similar expression pattern both in the cervical loop and in the inner enamel epithelium (Fig. 1A) and *Runx2* transcripts were also present in the mesenchyme, which was supported by PCR analysis (Fig. 1A). *Runx3* and *Cbfb* mRNA transcripts were also detected both in the dental epithelium and mesenchyme, which was supported by PCR analysis (Fig. 1A).

### The incisor phenotypes in Runx1 mutants

To analyze the functional role of *Runx1* in the dental epithelium of the growing incisors, we examined the incisal phenotypes of conditional knockout (*K14-Cre/Runx1*^fl/fl^) mice, in which *Runx1* expression was disrupted in the epithelium using Cre recombination^26^. The efficiency of the K14-Cre recombination of our mice was previously confirmed using Rosa26R reporter mice and X-gal staining^9^.

At P28, *Runx1* mutants demonstrated column-shaped incisors with a loss of transparency, while the control mice developed fine-edged incisors (Fig. 1B). A micro-CT analysis revealed that the *Runx1* mutant mice had abnormally shaped and extremely short incisors in comparison to the control mice (Fig. 1B). The labial surface of the mutant incisors was lacking enamel (Fig. 1B). On the other hand, there were no evident changes in the morphology of the mutant molars (Fig. 1B).

A histological analysis confirmed the shortening of the *Runx1* mutant incisors at P0 (Fig. 1C). Under higher magnification, the mutant cervical loop demonstrated hypoplasia with a remarkably reduced stellate reticulum region. In wild-type incisors, ameloblast precursors started to be polarized and their nuclei aligned at the apical side of the cells; such polarization was not observed in the *Runx1* mutant incisors (Fig. 1C). Enamel matrix was not evident on the labial surface of the Runx1 mutant incisors at E28 (Fig. 1D).

Double immunostaining of Ki67 and Keratin 14 (K14) revealed numerous proliferative cells in the epithelial layer in the cervical loop of control incisors. In contrast, the number of Ki67-immunoreactive proliferating epithelial cells was markedly reduced in the *Runx1* mutant incisors (Fig. 1E), indicating that the proliferative activity in the cervical loop was markedly disturbed by *Runx1* deficiency.

It is established that apoptotic cells are localized at the stellate reticulum of the cervical loop of the growing incisors^23^. TUNEL staining revealed that the number of apoptotic cells in the cervical loop was significantly decreased by *Runx1* deficiency (Fig. 1F).

### Downstream targets of Runx1 signaling in the incisors

**Figure 2:**
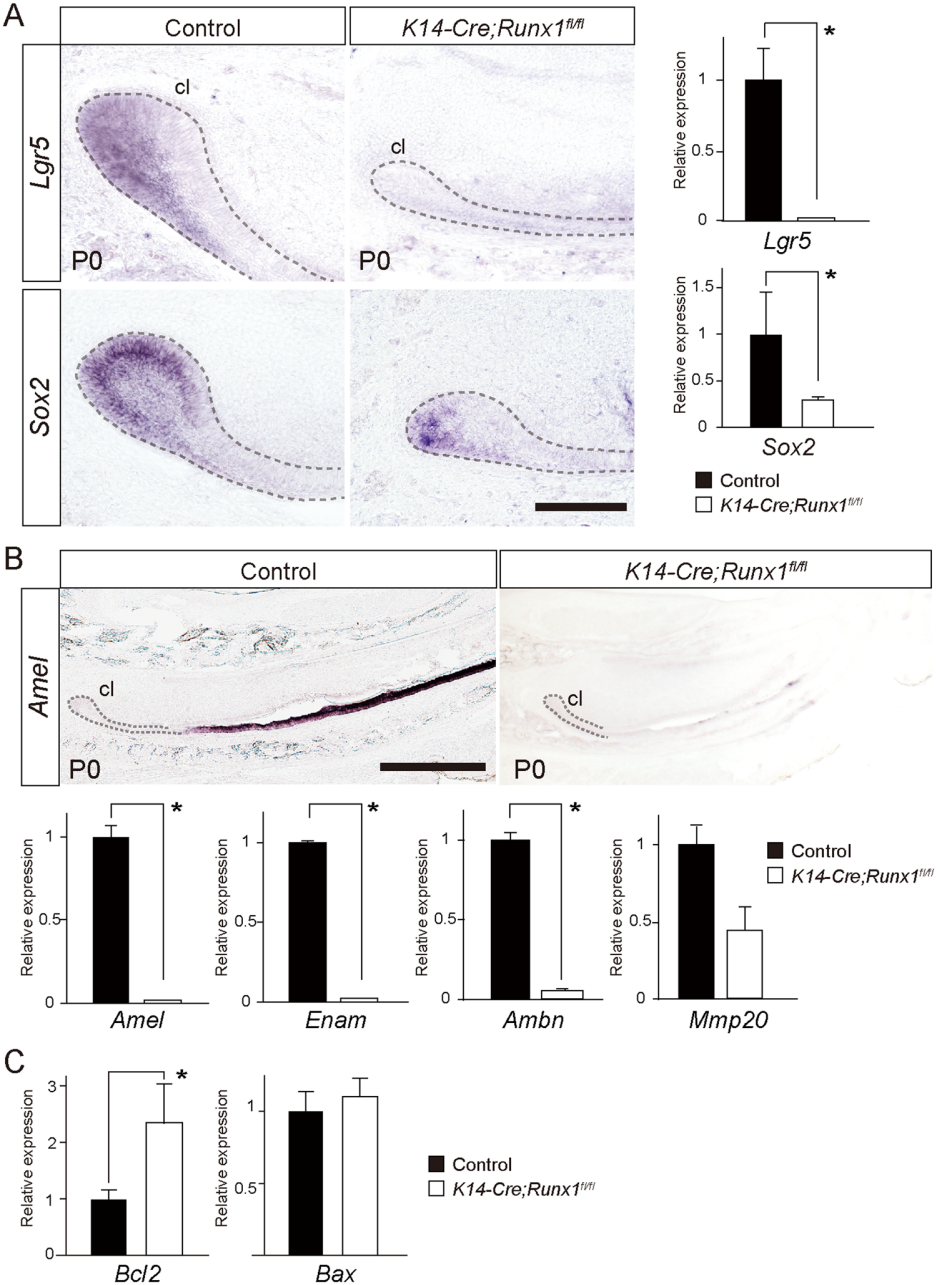
The downstream target molecules of Runx1 signaling in the growing incisors. (**A**) *In situ* hybridization analyses demonstrated that the expression of *Lgr5* and *Sox2* (general stem cell markers) was significantly downregulated in the *Runx1* mutants. A qPCR confirmed the downregulation of the *Lgr5* and *Sox2* expression in the mutants. *p < 0.05. Scale bar: 100 μm. (**B**) An *in situ* hybridization analysis demonstrated that the expression of *Amelogenin (Amel)*, an enamel matrix protein, was significantly downregulated in the *Runx1* mutants. A qPCR also confirmed the significant downregulation of *Amel*, *Amelogenin (Amel), Enamelin (Enam), Ameloblastin (Ambn)*. The expression of *Mmp20* was not significantly affected. *p < 0.05. Scale bar: 500 μm. (**C**) A qPCR A demonstrated that the *Bcl-2* expression was upregulated in the *Runx1* mutant cervical loop. Bax was not affected. *p < 0.05. cl, cervical loop.

To investigate how *Runx1* deficiency leads to the defective incisor growth, we evaluated the possible downstream target genes by *in situ* hybridization and a qPCR. It is established that *Lgr5 and Sox2* are specific markers of the epithelial stem cells in the cervical loop in the continuously growing mouse incisor^2,27^. In the present study, *in situ* hybridization and qPCR revealed that the expression levels of both *Lgr5* and *Sox2* were downregulated in the *Runx1* mutant incisors and the downregulation of *Lgr5* was more pronounced than that of *Sox2* (Fig. 2A). The present *in situ* hybridization analysis and qPCR also demonstrated the significant downregulation of *Amelogenin (Amel), Enamelin (Enam), Ameloblastin (Ambn)* in the *Runx1* mutant incisors (Fig. 2B). *Mmp20* expression was not deviated significantly (Fig. 2B).

In order to evaluate the possible cause of decreases in apoptotic cells at the labial side of the cervical loop, we evaluated the *Bcl-2* and *Bax* expression. qPCR of the cervical loop showed that anti-apoptotic molecule *Bcl-2* was upregulated in the *Runx1* mutant cervical loop (Fig. 2C). Indeed, such upregulation of *Bcl-2* due to Runx1 deficiency has also been observed in hematopoietic stem cells^28^

### Stat3 phosphorylation in Runx1 mutant incisors

Runx1 acts as a tumor promoter in the formation and maintenance of mouse skin cancer through its promotion of Stat3 activation^15,29^ and Runx1 stimulates the Stat pathway through the repression of suppressor of cytokine signaling 3 (Socs3), a major negative feedback regulator of Stat3-activating cytokines^13^. This Stat3/Socs3 pathway plays a key role in promoting hair cell regeneration through stem cell activation, cell division, and differentiation in zebrafish^30^. Hence, we assumed that *Runx1* deficiency might affect Stat3 signaling in the pathogenesis of defective phenotypes in the *Runx1* mutant incisor.

**Figure 3:**
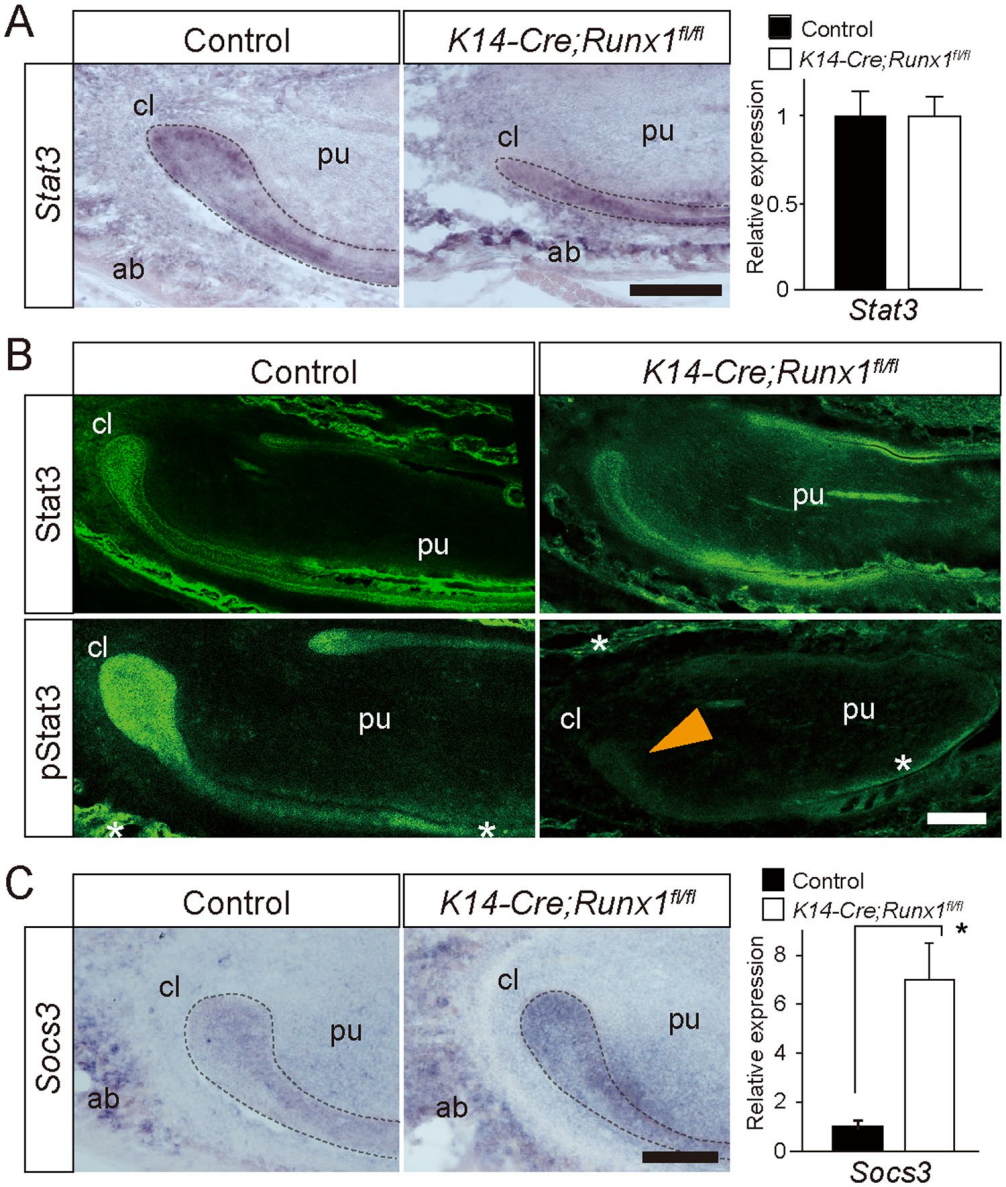
(**A**) An *in situ* hybridization analysis demonstrated that the mRNA expression of *Stat3* was evident in the cervical loop and the alveolar bone in the Runx1 mutant and control mice. A qPCR demonstrated that the *Stat3* expression was not affected significantly by Runx1 deficiency. Scale bar: 200 μm. (**B**) Stat3 immunoreactivity was widely distributed in both the epithelium and the mesenchyme of the incisors and the surrounding bone. Stat3 immunoreactivity was not affected by *Runx1* deficiency. In contrast, the pStat3 immunoreactivity of the cervical loop epithelium and the differentiating ameloblast was intense in control mice. In the *Runx1* mutants, immunoreactivity to pStat3 was remarkably disturbed at the cervical loop epithelium (arrowhead), but not in the odontoblast layers or on the alveolar bone surface (asterisks). Scale bar: 200 μm. (**C**) An *in situ* hybridization analysis demonstrated that the mRNA expression of *Socs3* was evident in the cervical loop and upregulated in Runx1 mutants. A qPCR demonstrated that the *Socs3* expression was significantly upregulated by Runx1 deficiency. Scale bar: 200 μm. *p < 0.05. cl, cervical loop; pu, dental pulp; ab, alveolar bone.

Our *in situ* hybridization analysis and qPCR showed that *Stat3* was expressed in the cervical region and was not affected by Runx1 deficiency (Fig. 3A).

The Stat3 activity was evaluated based on the state of phosphorylation. Stat3 immunohistochemistry demonstrated that the Stat3 immunoreactivity was widely detected in both the epithelium and the mesenchyme of the incisors and the surrounding bone (Fig. 3B). The spatial distribution of Stat3 immunoreactivity demonstrated similar distribution with regard to the expression of *Stat3* mRNA (Fig. 3B). Furthermore, the spatial distribution of Stat3 immunoreactivity was not deviated in *Runx* mutants, indicating that Runx1 deficiency did not affect Stat3 expression (Fig. 3B).

The distribution of phospho-Stat3 (pStat3) immunoreactivity was similar to that of Stat3 and immunoreactivity to pStat3 was specifically intense in the cervical loop (Fig. 3B). Interestingly, pStat3 immunoreactivity was specifically disturbed at the cervical loop in the *Runx1* mutants, whereas pStat3 immunoreactivity on the alveolar bone surface and odontoblasts were not affected (Fig. 3B). These findings indicated that Runx1 could regulate Stat3 activity specifically in the cervical loop epithelium through the modulation of Stat3 phosphorylation.

Our *in situ* hybridization analyses indicated the mRNA expression of *Socs3* in the cervical loop and the significant upregulation of *Socs3* expression in the *Runx1* mutant (Fig. 3C). Such upregulation of *Socs3* was also confirmed by qPCR (Fig. 3C). These findings suggested that Runx1 signaling affects Stat3 phosphorylation through, at least in part, the modulation of the *Socs3* expression.

### The inhibition of Stat3 phosphorylation in the growing incisors

**Figure 4:**
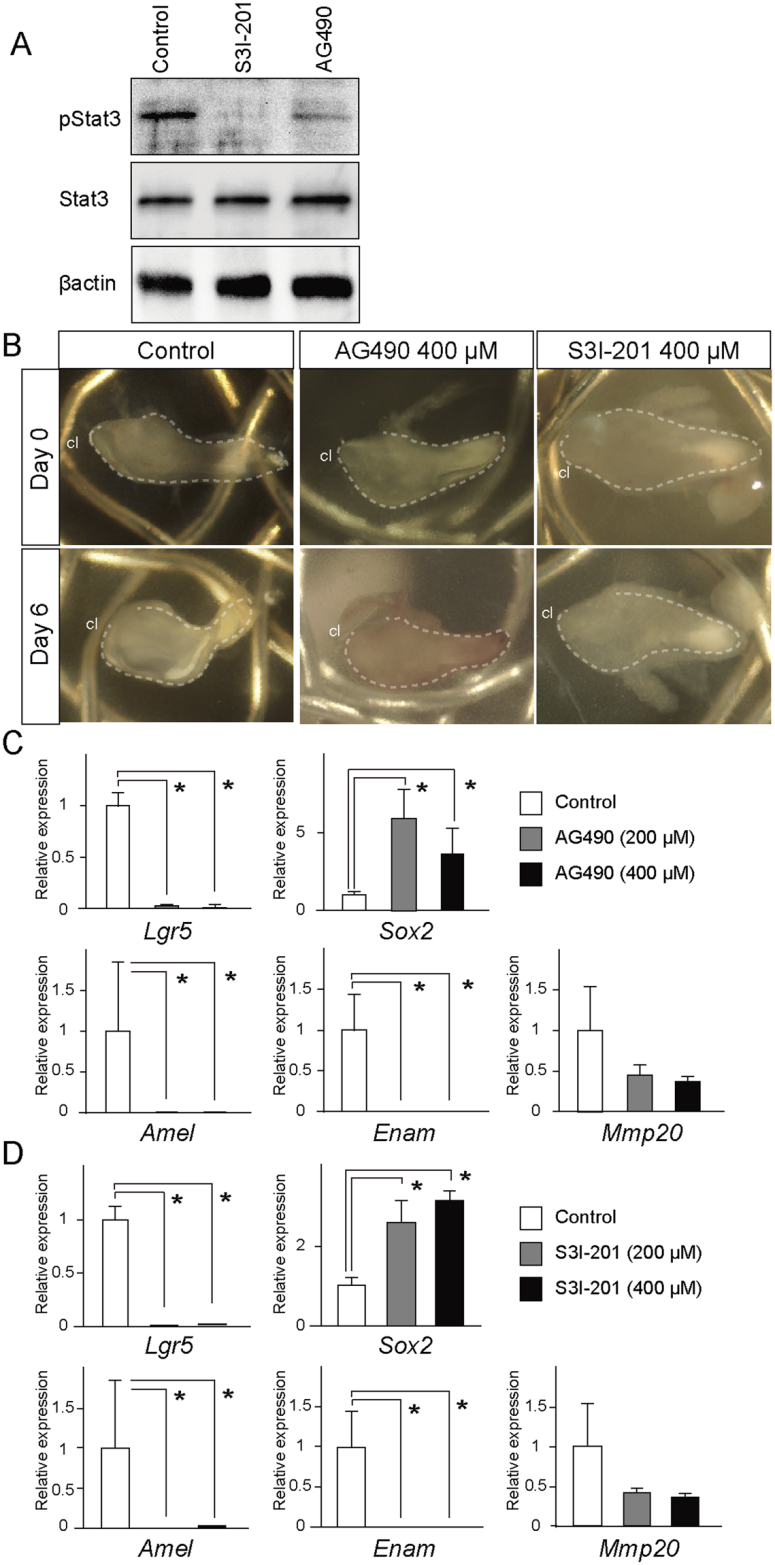
*In vitro* culture of the incisors and Stat3 inhibitor treatment. (**A**) A Western blot analysis showed that the application of Stat3 inhibitors of Ag490 or S3I-201 suppressed immunoreactivity to pStat3 but not to Stat3. (**B**) AG-490 or S3I-201, specific pharmaceutical Stat3 inhibitors, were applied on explanted lower incisors at E16.0 and cultured for 6 days. The size of the incisor explants did not change with Ag490 or S3I-201 treatment. (**C**) A qPCR analysis of the lower incisors showed that Ag490 treatment resulted in the marked downregulation of the *Lgr5* expression, while the expression of *Sox2* was upregulated. Ag490 treatment also resulted in the downregulation of the expression of *Amelogenin*, while AG490 significantly upregulated the *Socs3* expression. *p < 0.05. (**D**) The Treatment of S3I-201 also specifically downregulated the expression levels of *Lgr5* and *Amelogenin*, whereas the *Sox2* expression did not deviate and the Socs3 expression was upregulated by S3I-201 treatment. *p < 0.05. cl, cervical loop.

To evaluate the functional roles of Stat3 signaling, AG490, a selective Jak2/Stat3 inhibitor that prevents Stat3 phosphorylation, and S3I-201, a direct Stat3 inhibitor that blocks Stat3 dimerization and DNA-binding^31^ was applied to explanted lower incisors at E16.0 and cultured for 6 days. A Western blotting analysis confirmed that the application of the Stat3 inhibitors Ag490 or S3I-201 suppressed immunoreactivity to pStat3, while the Stat3 immunoreactivity was not affected (Fig. 4A). The scanned full blots are presented in Supplemental Fig. S5.

*In vitro* culture allowed for the continuous monitoring of the explants and the cervical loops in the incisor were translucent and visible during the culture period. After six days of culture, some explants became bent and marked elongation was not observed (Fig. 4B). Furthermore, our histological observation showed that some tissue damages was observed at the tip of the incisal explants (Fig. S2A); however, most of the tissue, including the cervical loop regions, showed no tissue degradation or deterioration of morphology. Ki67 immunohistochemistry further demonstrated that proliferative activity was retained in both the dental epithelium and mesenchyme of the explants after long-term culture (Fig. S2B). These finding indicate that incisor explants remained viable over the course of culture.

qPCR of the lower incisors showed that Ag490 treatment resulted in the marked downregulation of the *Lgr5* expression, whereas the expression of *Sox2* was significantly upregulated (Fig. 4C). Whole-mount *in situ* hybridization also confirmed the upregulated Sox2 expression in the cervical loop in the AG490-treated samples (Fig. S2C). Ag490 treatment also resulted in the downregulation of *Amelogenin* and *Enamelin*, whereas the expression of *Mmp20* was not significantly affected (Fig. 4C). Treatment of S3I-201 also specifically downregulated the expression of *Lgr5* and enamel matrix protein genes, whereas the expression of *Sox2* was upregulated (Fig. 4D). Treatment of S3I-201 also specifically downregulated the expression of *Amelogenin* and *Enamelin* (Fig. 4D). Taken together, these findings suggest that Stat3 activation is involved in the induction of *Lgr5* and the expression of enamel matrix protein genes.

### Rescue of the defective Runx1 mutant incisor phenotype by Stat3 activation

To confirm the role of Stat3 activation in the induction of the *Lgr5* and enamel matrix protein genes under Runx1 signaling, we evaluated the possibility of rescuing the defective *Runx1* mutant incisor phenotype, which showed the suppressed expression of *Lgr5*, by applying a pharmaceutical Stat3 activator to the mutant explants. A previous study demonstrated that ZnCl_2_ stimulation activates Stat3 phosphorylation and that activated Stat3 promotes embryonic stem cell pluripotency through the upregulation of genes such as *Oct4* and *Sox2*^32^.

**Figure 5:**
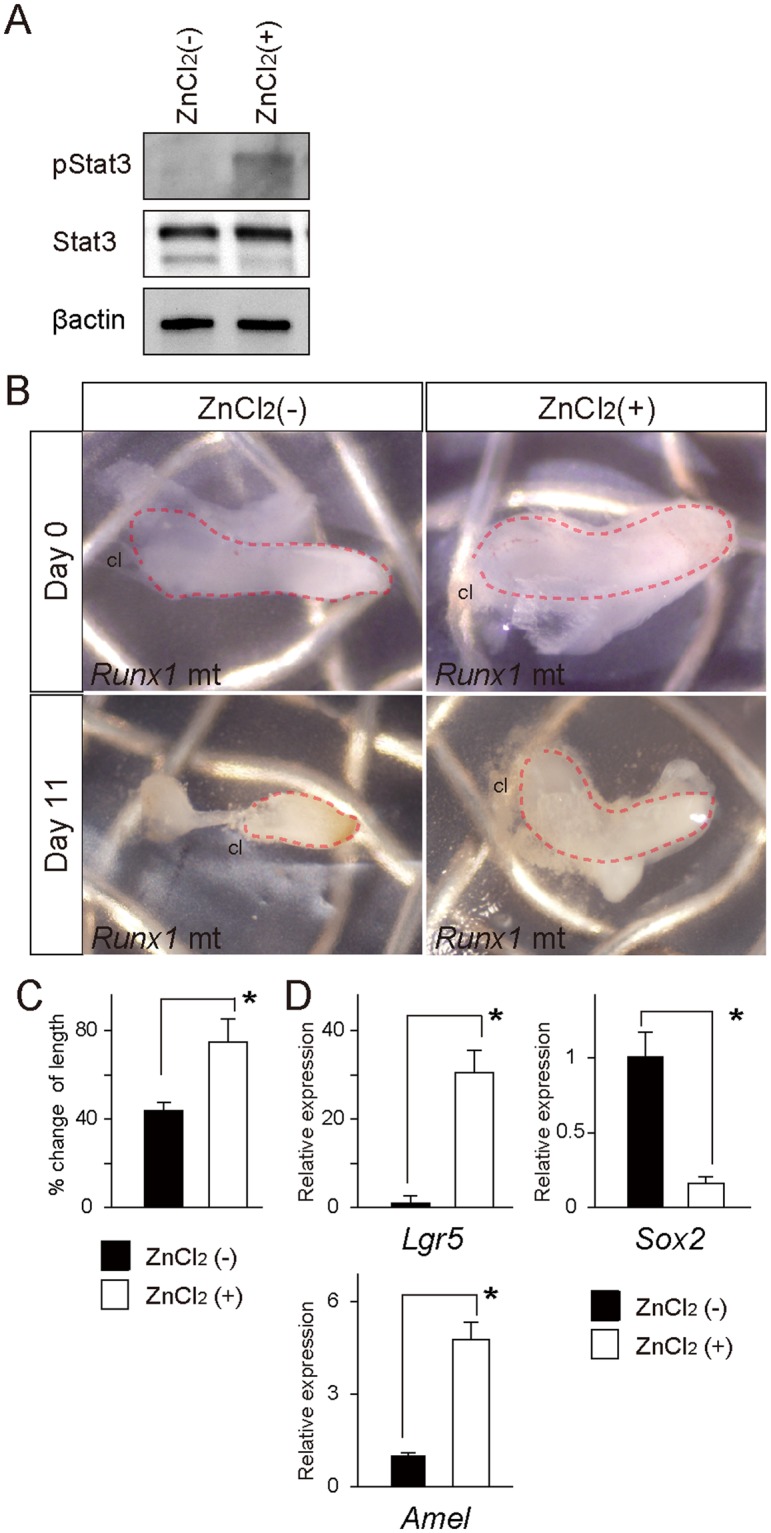
*In vitro* culture of the *Runx1* mutant incisors and ZnCl_2_ treatment (**A**) A Western blot analysis showed that the application of Stat3 activator of ZnCl_2_ upregulated immunoreactivity to pStat3, but not to Stat3. (**B**) After 11 days of culture, the *Runx1* mutant incisor explants became shrunk and bent, while the ZnCl_2_-treated *Runx1* mutants were bigger than the mutants without ZnCl_2_-treatment. (**C**) The percent-change in the length of the explants revealed that ZnCl_2_ treatment rescued the shrinkage of the mutant incisors. *p < 0.05. (**D**) A qPCR of the *Runx1* mutant incisors demonstrated that ZnCl_2_ treatment resulted in increases in the *Lgr5* expression, while expression of *Sox2* was downregulated. ZnCl_2_ treatment also rescued the downregulated expression of *Amelogenin* and downregulated *the Socs3* expression. *p < 0.05. cl, cervical loop.

The Western blotting analysis of the cervical loop in the present study confirmed that the application of the Stat3 activator ZnCl_2_ upregulated immunoreactivity to pStat3, while the Stat3 immunoreactivity was not affected (Fig. 5A). The scanned full blots of four samples are presented in Supplemental Fig. S6.

In our organ culture, the *Runx1* mutant incisor explants shrank and became bent

after 11 days of culture (8/8 cases; Fig. 5B). Interestingly, ZnCl_2_ treatment increased the length of the mutant explants markedly (8/8 cases; Fig. 5B). The percent-change in the length indicated that ZnCl_2_ partially rescued the developmental defects of the Runx1 mutants (Fig. 5C). Our histological observation revealed that tissue degradation was evident at the tip of the incisal explants (Fig. S3A), while the proximal region of the tissue, including the cervical loop regions, showed no tissue damage. Ki67 immunohistochemistry further demonstrated that the proliferative activity was retained in both the dental epithelium and the mesenchyme of the explants after long-term culture (Fig. S3B). These findings indicate that the incisor explants of the Runx1 mutants remained viable over the course of culture.

qPCR demonstrated that ZnCl_2_ treatment significantly upregulated the expression of *Lgr5* and *Amelogenin* (Fig. 5D). The expression of *Sox2* was downregulated by ZnCl_2_ treatment, while the expression of Socs3 was downregulated (Fig. 5D). These findings suggest that a Stat3 activator specifically rescued the suppressed *Lgr5* expression in the *Runx1* mutant incisor.

## Discussion

The Rodent incisors is a self-renewing organ that provides a remarkable example of a requirement for a continuous supply of progenitors from stem cells. The present results provide the first genetic evidence to support that *Runx1* has a specific function in regulation of the *Lgr5*-expressing epithelial stem cells in the growing incisors through Stat3 activation. Furthermore, defective phenotypes of *Runx1* mutant were rescued by pharmaceutical Stat3 activation, suggesting that Stat3 phosphorylation regulate the epithelial stem cell, which mediate the Runx1-Lgr5 axis to regulate the homeostasis of the continuous growth of the incisors.

The defective phenotype of the *Runx1* mutant incisor was similar to that of *Cbfb* mutants. Since Cbfb acts as a binding partner for all Runx proteins (Runx1, Runx2 and Runx3)^33^, the targeted inactivation of its expression abrogates the activity of all Runx complexes^34^. In wild-type mice, the expression of *Runx1* and *Runx2* overlapped in the enamel epithelium in the incisors and *Runx3* was also evident in the dental epithelium; hence, it was assumed that there might be redundancy among the functions of *Runx1, Runx2* and *Runx3*. In this study, the *Runx1* mutant phenotype was almost identical to the *Cbfb* mutant one, indicating that Runx1 has dominant roles in homeostasis and in the enamel formation of the growing incisors.

To assess how Runx1 signaling affects the epithelial stem cells in the growing incisors, we specifically examined the possible involvement of Stat3 signaling. It is established that Stat3 is essential in the maintenance of stem cells and cancer stem cells. The previous study demonstrated that Runx1 binds directly to the *Socs3* promoters to represses their transcription in keratinocytes, and ultimately upregulates Stat3 activity by enhancing phosphorylation^16^; hence, we assumed that the Stat3 activity might be affected in the *Runx1* mutant incisors. qPCR and *in situ* hybridization in the present study showed that the Socs3 expression was upregulated in association with *Runx1* depletion. These findings suggested that Socs3 might contribute—at least in part—to the regulation of Stat3 activity under the control of Runx1 signaling. The self-renewal of stem cells has a regulatory mechanism that is similar to that of the proliferation of cancer cells and STAT3 is an essential component of this mechanism in embryonic stem cells and various types of stem cells and cancer stem cells. Indeed, Runx1 stimulates Stat3 signaling in three epithelial cancer cell types through the transcriptional repression of a Stat3 inhibitor, SOCS3, which triggers cancer cell growth. Although we did not identify the specific kinase or molecule that regulates the phosphorylation of Stat3 in Runx1 signaling, our findings on the Socs3 expression suggested that Runx1 might be involved directly or indirectly in the downregulation of the Socs3 expression, which might explain how Runx1 regulates the Stat3 phosphorylation.

*Lgr5* is an established marker of epithelial stem cells in the intestinal crypt and in the hair follicle bulge and germ, and was identified as a Wnt target gene in the intestinal stem cell niche^19,35,36^. In the growing incisors, *Lgr5*-expressing cells were identified as actively cycling stem cells in the cervical regions; however, Wnt reporter was not present in the cervical loop epithelium, but was evident in the mesenchyme underlying the cervical loop. Immunostaining revealed that Stat3 is specifically activated in the cervical loop epithelium; thus, we explored the possible association between the Stat3 activity and the stem cell marker genes. It was of interest that *in vitro* culture with the Stat3-inhibitor Ag-490 or S3I-201 specifically suppressed the expression of *Lgr5*, but not *Sox2*. Furthermore, the reintroduction of Stat3 singling using ZnCl_2_, which activated Stat3 signaling, rescued the suppressed expression of *Lgr5* in the *Runx1* mutant incisors. These findings clearly indicated that Runx1 signaling could regulate the expression of *Lgr5*—at least in part—through the modification of Stat3 signaling in the cervical regions. The induction of *Lgr5* by the activation of Stat3 is also observed in the progression of skin-related basal cell carcinomas^37^. On the other hand, the *Runx1* mutant mice showed the downregulated expression of *Sox2*, as well as the expression of *Lgr5*. It is likely that *Runx1* deficiency might also affect regulatory pathways other than the Stat3 signaling pathway, which could influence the stem cells in the cervical loop.

Mice harboring an epidermal-specific constitutive Stat3 deletion were generated using K5-Cre mice. This mouse demonstrated defective wound healing, hair cycling and thymocyte survival; however, tooth phenotypes has not been reported. It is possible that other Stat family might also contribute the homeostasis of the growing incisors and have functional redundancy with Stat3. Another possible explanation is related to the difference in the manner of Stat3 deletion. In the previous study using *stat3* mutants, the Cre-mediated deletion of the *Stat3* gene was driven by the *Keratin5 (K5)* promoter. *K14*-Cre is another well-known Cre driver that is used to generate conditional null mutant mice, and it was of interest that the Dicer null mutants driven by *K14* and K5, respectively, do not exhibit identical appendage phenotypes. Indeed, *K14-Dicer* mutants exhibited more severe phenotypes at birth than *K5-Dicer* mutants and this difference could be due to the earlier onset of the *K14-Cre* driver^38^, or more efficient deletion using the *K14* promoter. It is possible that the *K14-Cre* driver is appropriate for evaluating the function of Stat3 in the growing incisors. We are currently investigating the incisal phenotypes using *K14-Cre* driven *Stat3* conditional null mice.

**Figure 6:**
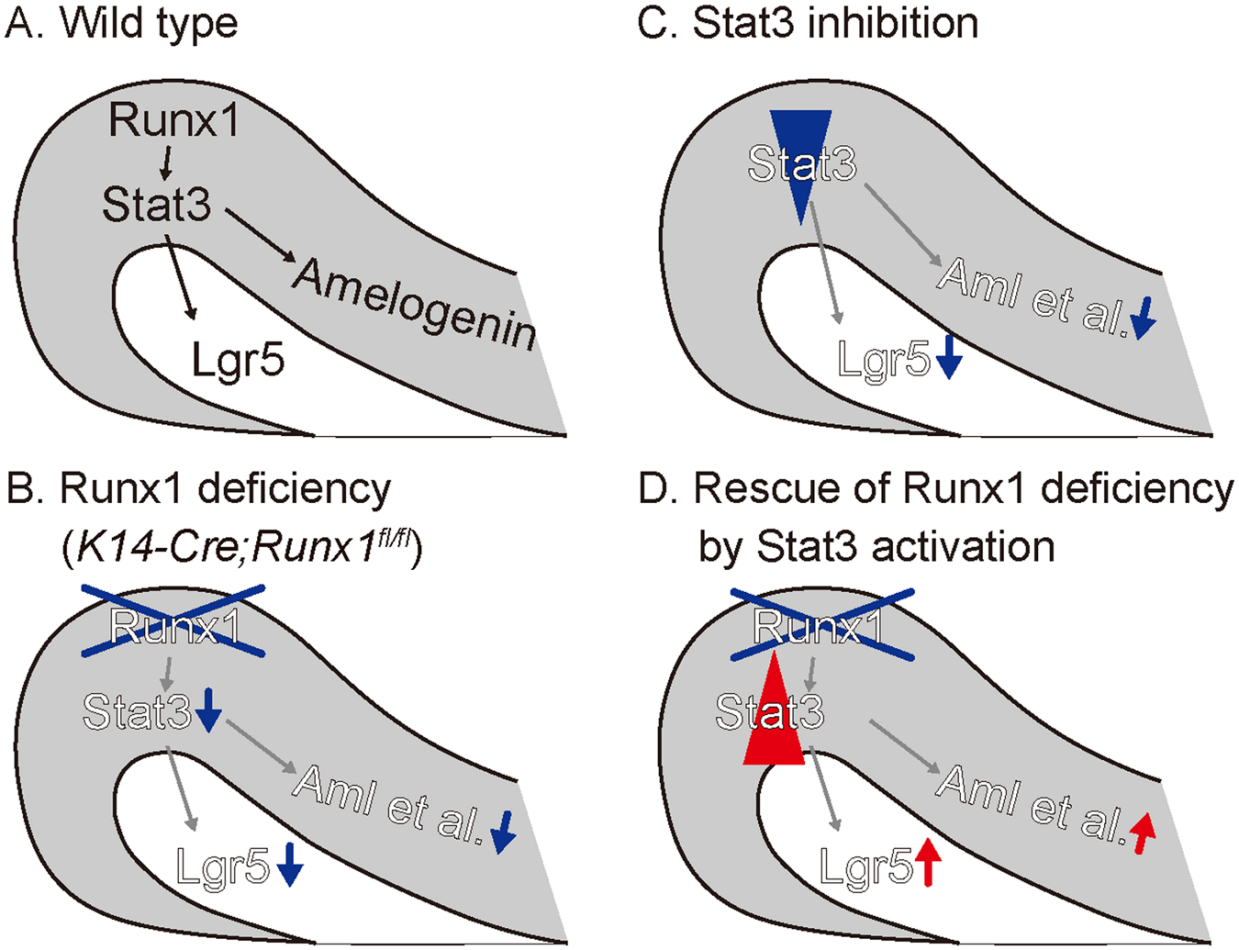
A schematic illustration of the key findings of this article. (**A**) In the cervical loop of the wild type incisors, Runx1 regulates Stat3 phosphorylation, which further regulates the expression of *Lgr5* and enamel matrix protein. (**B**) In the *Runx1* mutant incisors, Stat3 phosphorylation was significantly disturbed with the downregulated expression of *Lgr5* and enamel matrix proteins. (**C**) The pharmaceutical Inhibition of Stat3 signaling resulted in the significant downregulation of *Lgr5* and enamel matrix proteins. (**D**) Pharmaceutical Stat3 activation upregulated the expression *Lgr5* and enamel matrix proteins in Runx1 mutants.

In conclusion, we found that Runx1 is involved in the regulation of *Lgr5*-positive epithelial stem cells that differentiate into ameloblasts, through regulation of phosphorylation of Stat3 in the cervical loop of the growing incisors. Furthermore, defective phenotypes of *Runx1* mutants were rescued by treatment with a Stat3 activator, suggesting that Stat3 phosphorylation regulates epithelial stem cell, which mediate the Runx1-Lgr5 axis to regulate the homeostasis of the continuous growth of the incisors. Collectively, our study reveals an essential role for Runx1 and Stat3 activation epithelial stem cell function in the growing incisors (Fig. 6).

## Materials and Methods

### Animals

All of the animal experiments were performed in strict accordance with the guidelines of the animal care and use committee of the Osaka University Graduate School of Dentistry, Osaka, Japan. The protocol was approved by the Committee on the Ethics of Animal Experiments of Osaka University Graduate School of Dentistry. Mice were housed in the animal facility at the Department of Dentistry, Osaka University. Welfare guidelines and procedures were performed with the approval of the Osaka University Graduate School of Dentistry Animal Committee. C57/BL6 mice were used as wild-type (WT) mice. Mice expressing Cre recombinase under the control of keratin 14 promoter (K14-Cre; strain Tg(KRT14-cre)1Amc) were bred with floxed *Runx1* mice (Runx1tm3Spe) to produce epithelium-specific *Runx1*-knockout (*K14-Cre/Runx1*^*fl/fl*^) mice, as described previously^9,26^. We used their littermates or knockout embryos that did not carry the *K14-Cre/Runx1*^*fl/fl*^ genotype as controls. *K14-Cre/Runx1*^*fl/fl*^ mice were fed powder food.

### The histological analysis

The mouse embryonic heads were dissected in BGJb Medium (Gibco). This tissue was fixed in 4% paraformaldehyde at 4 °C overnight, equilibrated in graded sucrose, and embedded in Tissue-Tek (OCT compound, Sakura).

### The Micro-Computed Tomography (Micro-CT) Analysis

The lower incisors of the P28 control and conditional knockout mice were scanned with micro-CT (R_mCT2, Rigaku, Tokyo, Japan) at settings of 90 kV and 160 µA. The SimpleViewer software program (Rigaku) was used for the image analysis.

### The in situ hybridization (ISH) analysis

The digoxigenin-labeled RNA probes used in this study were prepared using a DIG RNA labeling kit (Roche) according to the manufacturer’s protocol using each cDNA clone as the template.

The probes were synthesized from fragments of *Runx1*, *Runx2*, *Runx3, Sox2, Lgr5, Stat3 and Amelogenin* (Allen Institute for Brain Science) and were amplified with T7 and SP6 adaptor primers through a PCR. After hybridization, the expression patterns for each mRNA were detected and visualized according to their immunoreactivity with anti-digoxigenin alkaline phosphatase-conjugated Fab fragments (Roche), as previously reported^9^. At least three embryos of each genotype were used for each analysis.

### Immunohistochemistry

After the removal of the OCT compound, immunofluorescence staining was performed on 20-µm sections using polyclonal rabbit-anti-Ki67 (1:400, ab15580, Abcam), monoclonal anti-mouse-K14 (1:200, ab7880, Abcam), monoclonal anti-rabbit-phospho-STAT3 (pStat3, 1:200, #9145, Cell Signaling Technology), monoclonal anti-mouse-STAT3 (Stat3, 1:200, #9139, Cell Signaling Technology) overnight at 4 °C. Then, Alexa488-conjugated donkey-anti-rabbit IgG (1:400, A21206, Molecular Probes) or Alexa546-conjugated goat-anti-mouse IgG (1:400, A11003, Molecular Probes) was used as a secondary antibody for 3 h at room temperature. The sections were then counterstained with DAPI (1:500, Dojindo) and mounted with fluorescent mounting medium (Dako). At least three embryos of each genotype were used for each analysis.

### Laser microdissection

The mandible was freshly embedded in OCT compound and frozen immediately. Tissues were serially sectioned at a thickness of 25 μm at −20 °C using a cryostat (CM 1950, Leica). The tissue sections were mounted thawed on a film-coated slide. We stained these slides with cresyl violet. Sections of the cervical loop and the neighboring incisal epithelial tissues were micro-dissected respectively (about 300 μm in length each) using a Leica Micro Laser System (LMD6500; Leica) and collected in a tube, as shown in Supplemental Fig. S1. Representative samples Pre- and Post-laser microdissection from sections of the cervical loop of the incisor are shown in Supplemental Fig. S1. Sections from each genotype were pooled for extraction of mRNA.

### Western blotting

The dissected tissues were lysed with RIPA buffer (nacalai tesque) supplemented with protease and phosphatase inhibitors (nacalai tesque). The lysates were centrifuged and the supernatant was heated in denaturing Laemmli buffer (Bio-rad Laboratories). Proteins were separated by SDS-PAGE and transferred to polyvinylidene difluoride membranes (Bio-rad Laboratories). The membranes were incubated with anti-Stat3 (1:1000, #9139, Cell Signaling Technology), anti-pStat3 (1:1000, #9145, Cell Signaling Technology), or beta-actin (1:2000, Sigma). The bound antibodies were detected with HRP-linked antibody (1:1,000, Cell Signaling Technology) and an ECL detection kit (Bio-rad Laboratories).

### RNA extraction and the real-time RT-PCR

We used the laser-microdissected tissues of the control and *K14-Cre/Runx1*^*fl/fl*^ mice to extract total RNA. Total RNA was isolated using ISOGEN (Nippon Gene) according to the manufacturer’s instructions. Total RNA was reverse transcribed to cDNA using an oligo (dT) with avian myeloblastosis virus reverse transcriptase (Takara). For the real-time RT-PCR, the cDNA was amplified with TaqDNA Polymerase (Toyobo) using a light cycler (Roche). The thermal profile for all SYBR Green PCRs was 95 °C for 30 s followed by 50 cycles of 3-step amplification, including 95 °C for 5 s, 55 °C for 10 s, 72 °C for 15 s, and a melting step, including 95 °C for 1 s, 73 °C for 15 s, 95 °C for 1 s, and 40 °C for 30 s for cooling. Data was normalized to *Gapdh* and expressed as relative value to the average of control group. The primer sequences are shown in Supplemental Table S1. At least three embryos of each genotype were used for each analysis.

### *In vitro* culturing of the incisors and Stat3 inhibitor treatment

Two different types of Stat3 inhibitors were applied in the *in vitro* cultures^39^. To exclude possible effects of serum on Stat3 signaling, the present tooth model was established using chemically-defined BGJB medium (Gibco) without serum supplementation. The lower incisor was dissected from the E16.0 embryo and cultured on a track-etched polycarbonate membrane filter (Nuclepore) in Trowell-type organ culture with serumless, chemically-defined medium (BGJB, Gibco) with or without AG490 (200–400 µM; Sigma-Aldrich) or Stat3 Inhibitor VI, S3I-201 (200–400 µM; Sigma-Aldrich). Trowell-type culture enables the long-term culture of tooth germ explants, and previous *in vitro* culture has shown that the explanted tooth germs remained viable for four weeks using serumless chemically-defined BGJB medium^40^. Tissues were harvested after 6 days of culturing, then processed for a qPCR and Western blotting.

### *In vitro* culture of the Runx1 mutant incisors and ZnCl2 treatment

To activate the phosphorylation of Stat3, ZnCl_2_ was applied in the *in vitro* cultures. The lower incisor of the *Runx1* mutant was dissected from the E16.0 embryo and cultured on a track-etched polycarbonate membrane filter (Nuclepore) in Trowell type organ culture with serumless, chemically-defined medium (BGJb, Gibco) with or without ZnCl_2_ (Sigma-Aldrich). The tissues were harvested after 11 days of culturing, then processed for a qPCR and Western blotting.

In addition, in order to quantify the morphological changes associated with ZnCl_2_ treatment in *Runx1* mutants, we measured the length of the incisal explants 11 days after culture. Since the explants became bent after long-term culture, the curved incisor length was traced and measured at the midpoint of the labial and lingual surface of the specimens using ImageJ software (NIH). The percent-change in the length of the explants was calculated to quantify the morphological changes associated with ZnCl_2_ treatment.

## Electronic supplementary material

Supplemental Table and Figures

## References

[1] Harada H (1999). Localization of putative stem cells in dental epithelium and their association with Notch and FGF signaling. J Cell Biol.

[2] Juuri E (2012). Sox2+stem cells contribute to all epithelial lineages of the tooth via Sfrp5+progenitors. Dev Cell.

[3] Seidel K (2010). Hedgehog signaling regulates the generation of ameloblast progenitors in the continuously growing mouse incisor. Development.

[4] Kuang-Hsien HJ, Mushegyan V, Klein OD (2014). On the cutting edge of organ renewal: Identification, regulation, and evolution of incisor stem cells. Genesis.

[5] Mitsiadis TA, Barrandon O, Rochat A, Barrandon Y, De Bari C (2007). Stem cell niches in mammals. Exp Cell Res.

[6] Mangan JK, Speck NA (2011). RUNX1 mutations in clonal myeloid disorders: from conventional cytogenetics to next generation sequencing, a story 40 years in the making. Crit Rev Oncog.

[7] Osorio KM, Lilja KC, Tumbar T (2011). Runx1 modulates adult hair follicle stem cell emergence and maintenance from distinct embryonic skin compartments. J Cell Biol.

[8] Raveh E (2006). Dynamic expression of Runx1 in skin affects hair structure. Mech Dev.

[9] Kurosaka H (2011). Core binding factor beta functions in the maintenance of stem cells and orchestrates continuous proliferation and differentiation in mouse incisors. Stem Cells.

[10] Huang G (2001). Dimerization with PEBP2beta protects RUNX1/AML1 from ubiquitin-proteasome-mediated degradation. EMBO J.

[11] Rawlings JS, Rosler KM, Harrison DA (2004). The JAK/STAT signaling pathway. J Cell Sci.

[12] Reich NC (2013). STATs get their move on. JAKSTAT.

[13] Cartwright P (2005). LIF/STAT3 controls ES cell self-renewal and pluripotency by a Myc-dependent mechanism. Development.

[14] Sano S (1999). Keratinocyte-specific ablation of Stat3 exhibits impaired skin remodeling, but does not affect skin morphogenesis. EMBO J.

[15] Osorio KM (2008). Runx1 modulates developmental, but not injury-driven, hair follicle stem cell activation. Development.

[16] Scheitz CJ, Lee TS, McDermitt DJ, Tumbar T (2012). Defining a tissue stem cell-driven Runx1/Stat3 signalling axis in epithelial cancer. EMBO J.

[17] Diaz N (2006). Activation of stat3 in primary tumors from high-risk breast cancer patients is associated with elevated levels of activated SRC and survivin expression. Clin Cancer Res.

[18] Baker BJ, Akhtar LN, Benveniste EN (2009). SOCS1 and SOCS3 in the control of CNS immunity. Trends Immunol.

[19] Van der Flier LG (2007). The Intestinal Wnt/TCF Signature. Gastroenterology.

[20] Harada H (2002). FGF10 maintains stem cell compartment in developing mouse incisors. Development.

[21] Parsa S (2010). Signaling by FGFR2b controls the regenerative capacity of adult mouse incisors. Development.

[22] Suomalainen M, Thesleff I (2010). Patterns of Wnt pathway activity in the mouse incisor indicate absence of Wnt/beta-catenin signaling in the epithelial stem cells. Dev Dyn.

[23] Yang Z, Balic A, Michon F, Juuri E, Thesleff I (2015). Mesenchymal Wnt/β-Catenin Signaling Controls Epithelial Stem Cell Homeostasis in Teeth by Inhibiting the Antiapoptotic Effect of Fgf10. Stem Cells.

[24] Chang JY (2013). Fibroblast growth factor signaling is essential for self-renewal of dental epithelial stem cells. J Biol Chem.

[25] Yamashiro T, Aberg T, Levanon D, Groner Y, Thesleff I (2002). Expression of Runx1, -2 and −3 during tooth, palate and craniofacial bone development. Mech Dev.

[26] Ono Minagi H (2017). Runx1 mediates the development of the granular convoluted tubules in the submandibular glands. PLoS One.

[27] Chang JY (2013). Self-renewal and multilineage differentiation of mouse dental epithelial stem cells. Stem Cell Res.

[28] Motoda L (2007). Runx1 protects hematopoietic stem/progenitor cells from oncogenic insult. Stem Cells.

[29] Hoi CS (2010). Runx1 directly promotes proliferation of hair follicle stem cells and epithelial tumor formation in mouse skin. Mol Cell Biol.

[30] Liang J (2012). The stat3/socs3a pathway is a key regulator of hair cell regeneration in zebrafish. [corrected]. J Neurosci.

[31] Siddiquee K (2007). Selective chemical probe inhibitor of Stat3, identified through structure-based virtual screening, induces antitumor activity. Proc Natl Acad Sci USA.

[32] Hu J (2016). Zinc Chloride Transiently Maintains Mouse Embryonic Stem Cell Pluripotency by Activating Stat3 Signaling. PLoS One.

[33] Levanon D (1994). AML1, AML2, and AML3, the human members of the runt domain gene-family: cDNA structure, expression, and chromosomal localization. Genomics.

[34] Wang Q (1996). The CBFbeta subunit is essential for CBFalpha2 (AML1) function *in vivo*. Cell.

[35] Barker N (2007). Identification of stem cells in small intestine and colon by marker gene Lgr5. Nature.

[36] Jaks V (2008). Lgr5 marks cycling, yet long-lived, hair follicle stem cells. Nat Genet.

[37] Jia J (2016). LGR5 expression is controled by IKKα in basal cell carcinoma through activating STAT3 signaling pathway. Oncotarget.

[38] Lyle S (2014). Dicer cooperates with p53 to suppress DNA damage and skin carcinogenesis in mice. PLoS One.

[39] Gurbuz V (2014). Effects of AG490 and S3I-201 on regulation of the JAK/STAT3 signaling pathway in relation to angiogenesis in TRAIL-resistant prostate cancer cells *in vitro*. Oncol Lett.

[40] Slavkin HC (1990). Cartilage, bone and tooth induction during early embryonic mouse mandibular morphogenesis using serumless, chemically-defined medium. Connect Tissue Res.

