# Supplementary material for: Runx1-Stat3 signaling regulates the epithelial stem cells in continuously growing incisors
Source: Sci Rep. 2018 Jul 19;8:10906. doi: 10.1038/s41598-018-29317-6 (PMC6053438; doi:10.1038/s41598-018-29317-6)
Supplement: Supplementary file 1 — Supplemental Table and Figures [file 41598_2018_29317_MOESM1_ESM.pdf]

Runx1-Stat3 signaling regulate the epithelial stem cells in continuously growing incisors.

Safiye E. Sarper[1], Toshihiro Inubushi[1], Hiroshi Kurosaka[1], Hitomi Ono [2], Koh-ichi Kuremoto[3], Takayoshi Sakai[2], Ichiro Taniuchi[4], Takashi Yamashiro[1]*.

[1]Department of Orthodontics and Dentofacial Orthopedics, Osaka University Graduate School of Dentistry, Osaka, Japan
[2]Department of Oral-facial Disorders, Osaka University Graduate School of Dentistry, Osaka, Japan
[3]Department of Advanced Prosthodontics, Graduate School of Biomedical & Health Sciences, Hiroshima University, Hiroshima, Japan
[4]Laboratory for Transcriptional Regulation, RIKEN Research Center for Allergy and Immunology, Yokohama, Japan

*Author for correspondence:
Takashi Yamashiro, DDS, PhD
Professor
Department of Orthodontics and Dentofacial Orthopedics, Graduate School of Dentistry, Osaka University
1-8 Yamada-Oka, Suita, OSAKA 565-0871, Japan
TEL : +81-6-6879-2958 FAX : +81-6-6879-2960
E-mail: yamashiro@dent.osaka-u.ac.jp

Supplemental Table

(A) Primers for *in situ* hybridization

	Forward Primer	Reverse Primer
Runx1	AGCATGGTGGAGGTACTAGCTG	GCGCCGTAGTATAGATGGTAGG
Runx2	CGAGGCAAGAGTTTCACCTTG	CTCATCCATTCTGCCGCTAGA
Runx3	AACTTCCTCTGCTCCGTGCTG	GTTCATGAGGTTGCCTGCTGA
Cbfb	ATGGTATGGGTTGCCTGGAG	TCAGGCCATGAAACCAACTGC
Amel	ACCTCATCCTGGAAGCCC	TCTTCCCGCTTGGTCTTG
Stat3	GAGGCCCTCCCAACATCT	TTCATTCCAAAGGGCCAA
Lgr5	ACTCCCCTGTACATCTCTTCCA	ATCTCATCCAGAAACGGGTATG
Sox2	CAGCGCATGGACAGCTAC	GTCGGCATCACGGTTTTT

(B) Primers for qPCR

	Forward Primer	Reverse Primer
Gapdh	AACTTTGGCATTGTGGAAGG	ACACATTGGGGGTAGGAACA
Lgr5	CGGGACCTTGAAGATTTCCT	GATTCGGATCAGCCAGCTAC
Sox2	GCACATGAACGGCTGGAGCAACG	TGCTGCGAGTAGGACATGCTGTAGG
Amel	TCCTGGAAGCCCTGGTTATATC	GTTGAGACAGCACAGGGATG
Enam	GGAACCACCAAATGAAGCAG	CCAAAGCCGTGATATCCAAA
Ambn	TTCCCATGGATAGGACCAAG	ATCAGCTCTCCTTCCTGCAA
Mmp20	AGCAAGAGAGGAGATGAAGGTGCT	AAGGTGGTAGTTGCTCCTGAAGGT
Socs3	CAAGAACCTACGCATCCAGTG	CCAGCTTGAGTACACAGTCGAA
Stat3	CTTGTCTACCTCTACCCCGACAT	GATCCATGTCAAACGTGAGCG
Bcl-2	TGAGTACCTGAACCGGCATCT	GCATCCCAGCCTCCGTTAT
Bax	CCCGAGAGGTCTTTTTCC	GCCTTGAGCACCAGTTTG

Supplemental Figure S1

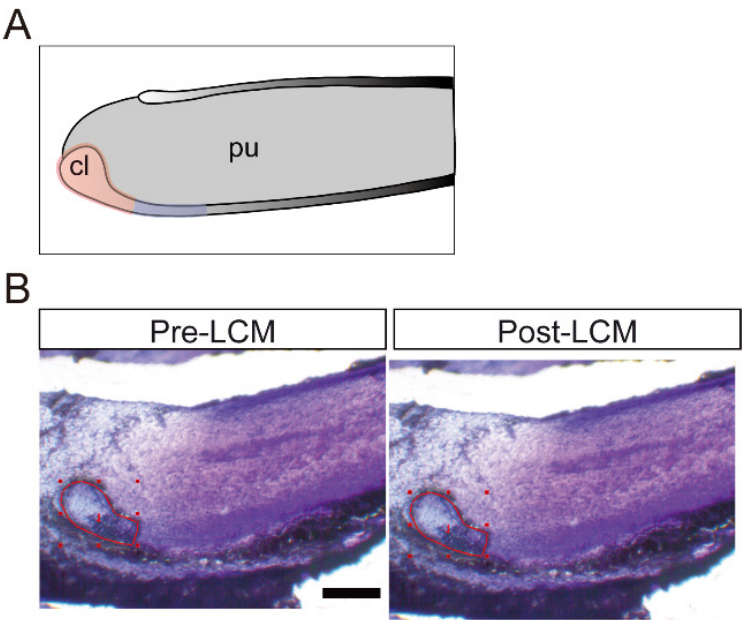

Supplemental Figure S1. (A) A schematic representation of the dissecting region of the cervical loop and the neighboring incisal epithelium. (B) Representative samples Pre- and Post-laser microdissection from sections of the cervical loop. Bar=200 μm. cl, cervical loop; pu, pulp.

Supplemental Figure S2

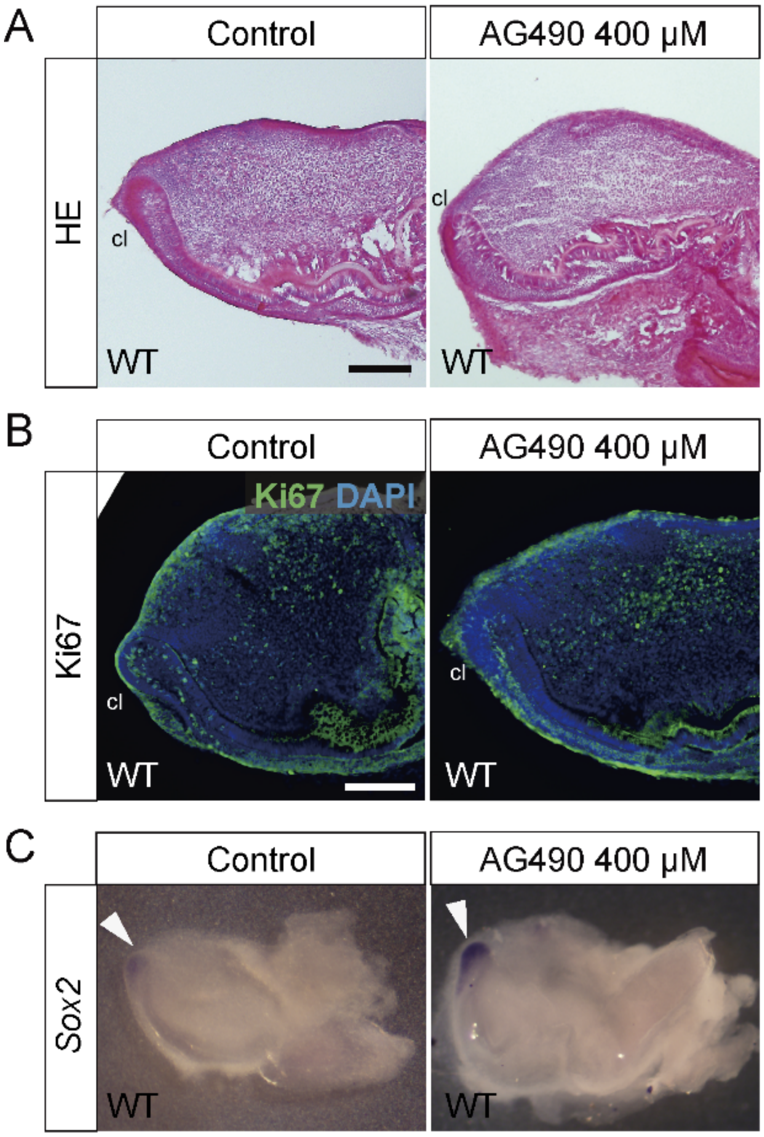

Supplemental Figure S2. *in vitro* culture of the incisors and Stat3 inhibitor treatment. (A) HE staining and (B) immunostaining for Ki67(green) demonstrated that explanted tooth germs remained viable over six days of organ culture. Scale bar: 200 μm. (C) Whole-mount in situ hybridization showed that *Sox2* expression was evident in the cervical loop and upregulated in the AG490 treated samples. Arrowheads: Sox2 expression in the cervical loop.

Supplemental Figure S3

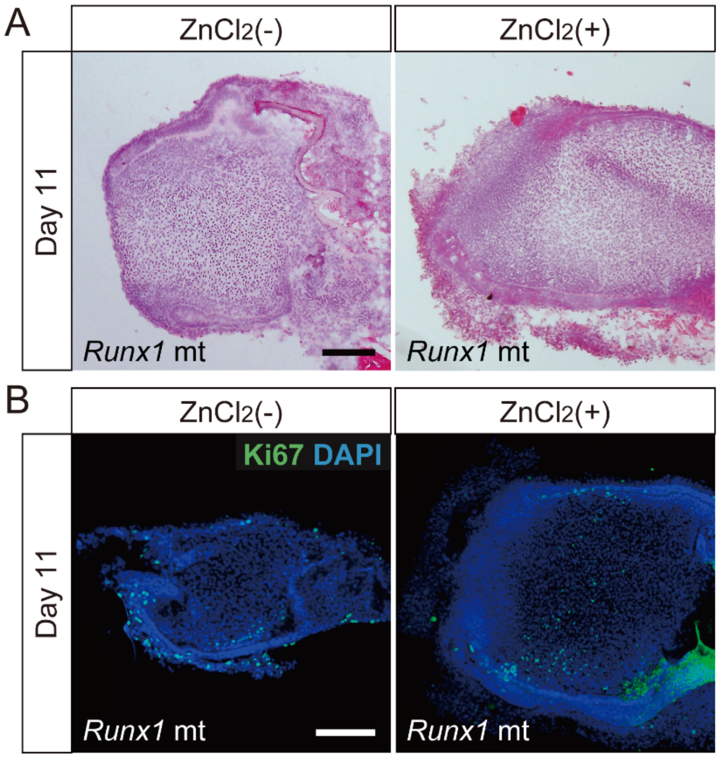

Supplemental Figure S3. In vitro culture of the *Runx1* mutant incisors and $ZnCl_2$ treatment. (A) HE staining and (B) immunostaining for Ki67(green) showed that the explanted tooth germs remained viable over 11 days of organ culture. Scale bar: 200 μm. Nuclei were counterstained with DAPI (blue).

Supplemental Figure S4

Epithelium

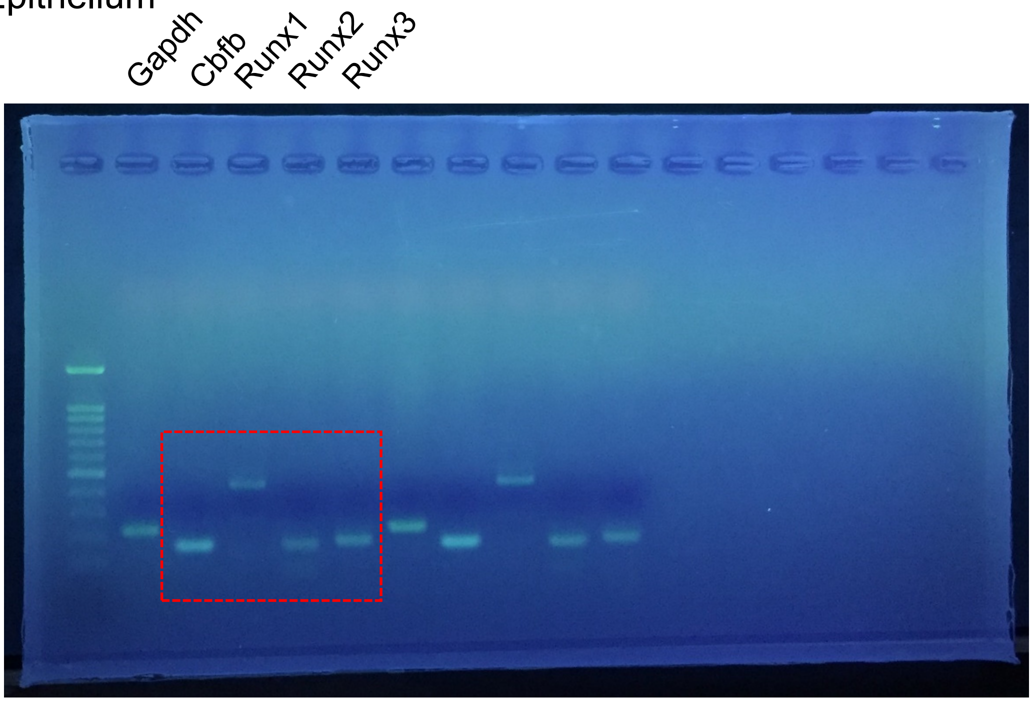

Mesenchyme

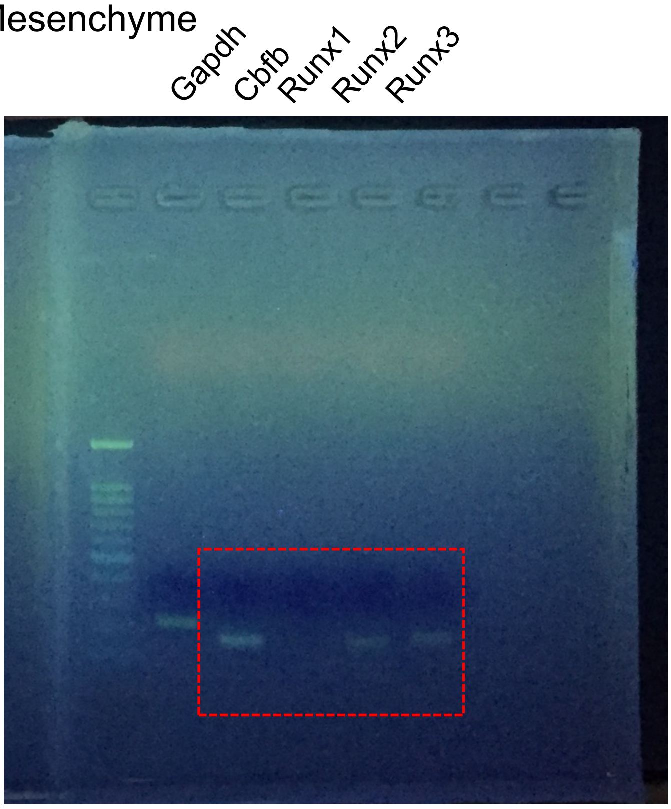

Supplemental Figure S4. Full-length PCR products from the dissected epithelium and mesenchyme in Figure 1A. The red, dotted lines show the cropping locations.

Supplemental Figure S5

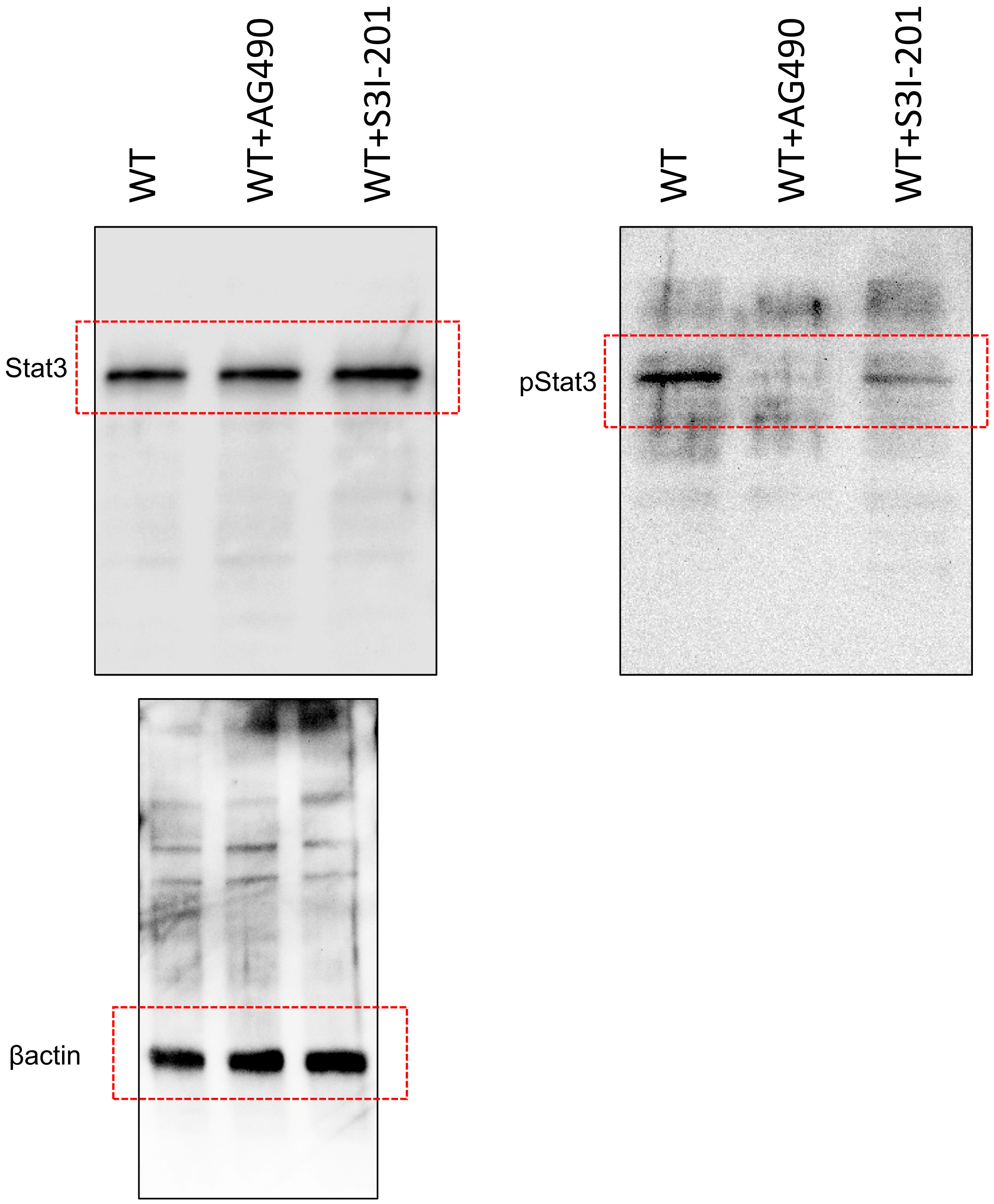

Supplementary Figure S5. Full-length blots of Stat3, pStat3 and βactin in the primary palatal tissue with or without Stat3 inhibitors (shown as cropped images in Figure 4A).

Supplemental Figure S6

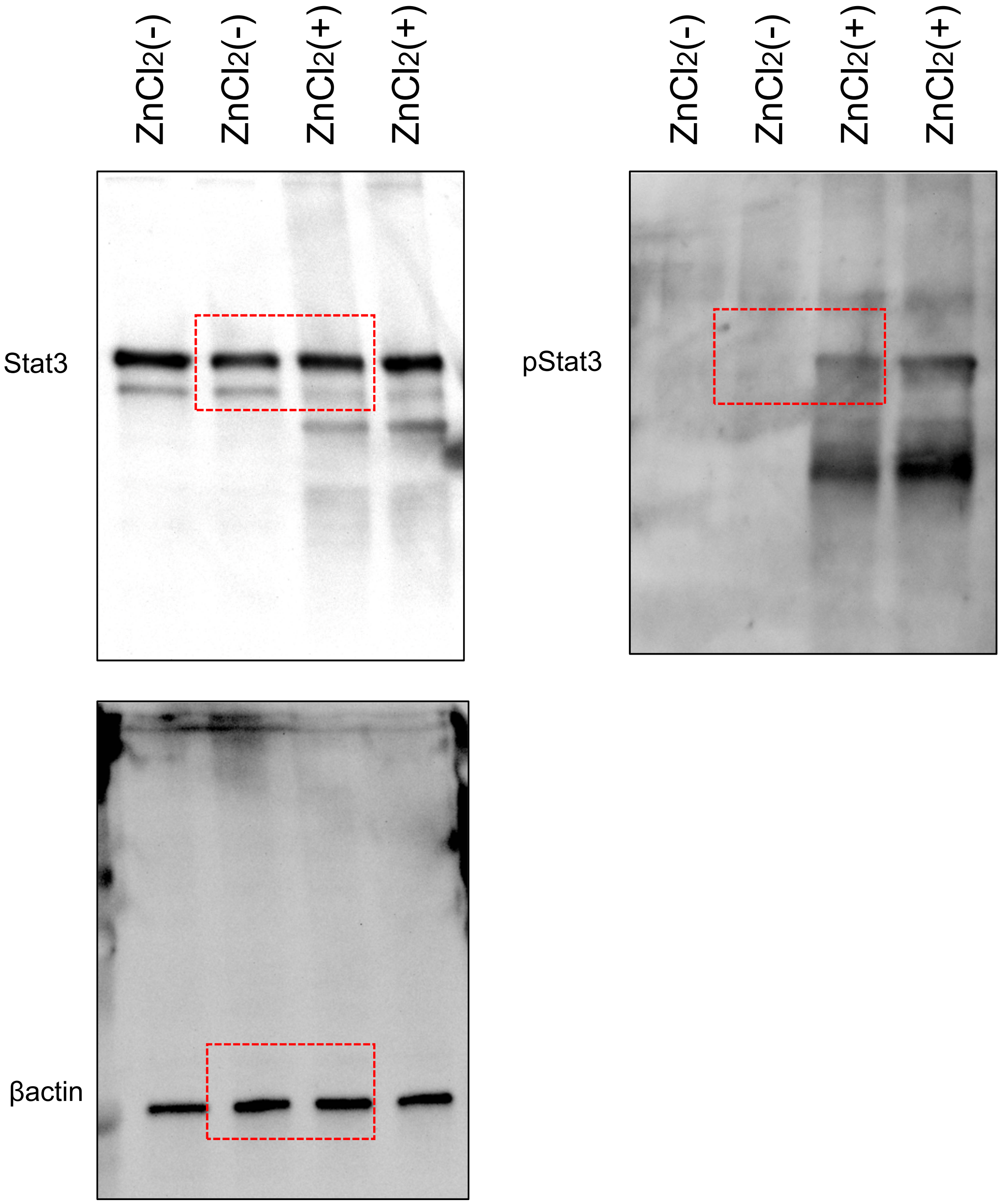

Supplementary Figure S6. Full-length blots of Stat3, pStat3 and βactin in the primary palatal tissue with or without Stat3 inhibitors (shown as cropped images in Figure 5A).
